# ﻿MycoKeys issue 100: progress and innovation to enhance rapid publication in fungal systematics

**DOI:** 10.3897/mycokeys.100.115344

**Published:** 2023-11-13

**Authors:** H. Thorsten Lumbsch, Pier Luigi Nimis, Dominik Begerow, Pavel Stoev, Lyubomir Penev

**Affiliations:** 1 The Field Museum, Chicago, USA The Field Museum Chicago United States of America; 2 University of Trieste, Trieste, Italy University of Trieste Trieste Italy; 3 University of Hamburg, Hamburg, Germany University of Hamburg Hamburg Germany; 4 Pensoft Publishers, Sofia, Bulgaria Pensoft Publishers Sofia Bulgaria

Since its inception in 2011 (Lumbsch et al. 2011), MycoKeys has published over 550 articles that have been cited more than 6000 times according to the Web of Science. Twelve years since its launch, and eight years since receiving its first Journal Impact Factor, we are now publishing the journal’s 100^th^ issue. This was only made possible by the high quality of submissions from authors who chose the journal as a vehicle to publish their results, the team of subject editors, numerous reviewers, and the efficient editing and publishing of the journal. This issue is a great occasion to look back and evaluate the performance of MycoKeys.

MycoKeys started with only 13 submissions in 2011, whereas the number of submitted manuscripts has been above 130 annually for the past six years (Fig. 1). Similarly, the number of published articles has grown, from 8 in the first year to above 50 annually in the last 6 years. To date, the journal has received a total of 1033 submissions and published 561 articles with an average acceptance rate of 55%. In recent times, the average time from submission to acceptance has been 70 days, and from acceptance to publication: 90 days. The number of article views has also increased to more than 450,000 annually for the last few years (Fig. 2). The articles address issues of systematics and taxonomy of all clades of the kingdom Fungi, however, the majority of papers deal with Ascomycota or Basidiomycota, including lichenized fungi.

**Figure 1.**
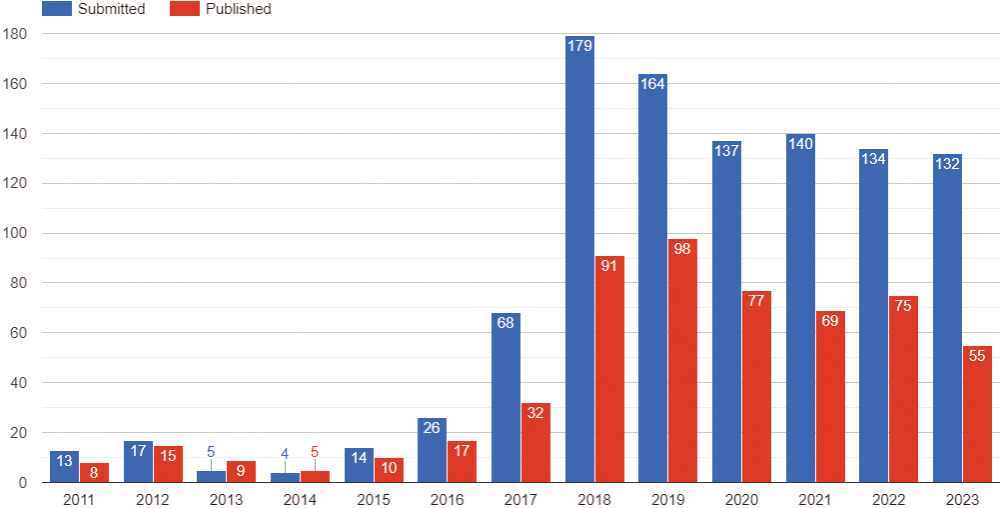
Submitted and published manuscripts in MycoKeys on a yearly basis since the launch of the journal in 2011. Data retrieved on 30 October 2023.

**Figure 2.**
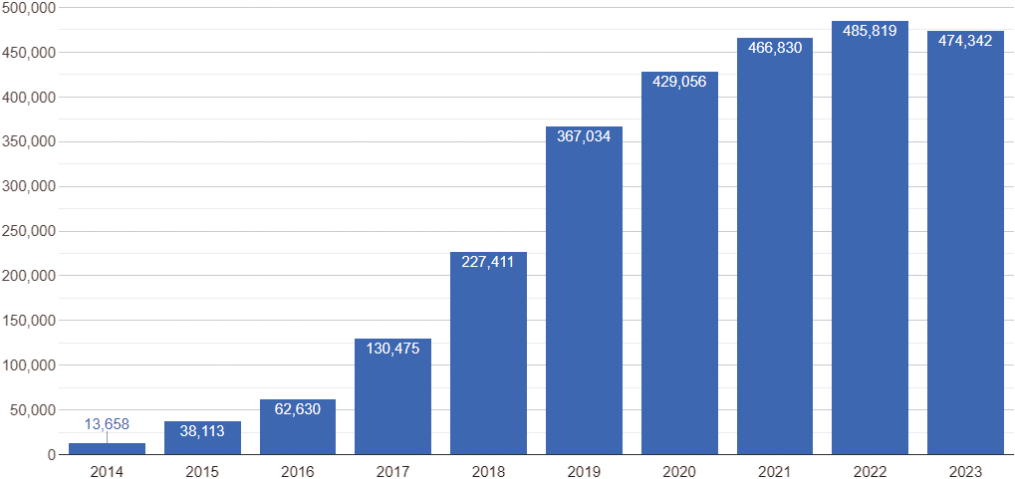
Article views for MycoKeys on a yearly basis since 2014. Data retrieved on 30 October 2023.

MycoKeys has attracted researchers from various parts of the world to publish their results (Fig. 3). Altogether, scientists from 80 countries have published in the journal to date. The greatest number of researchers come from China, Thailand, Germany, the United States of America, Sweden, and Italy.

**Figure 3.**
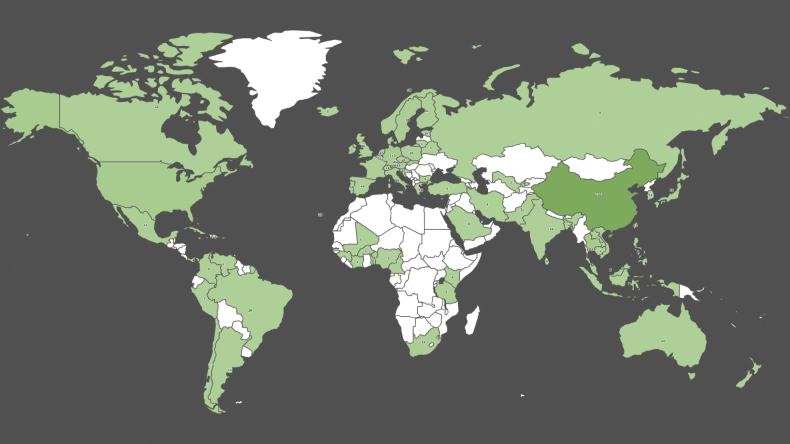
Authors in MycoKeys by country (all-time data). Data retrieved on 30 October 2023.

The top 10 most cited MycoKeys papers up until 31 October 2023 include papers addressing a wide array of issues, including: potential bias in the use of high throughput molecular identification methods (Tedersoo et al. 2015); quality control of generated sequences (Nilsson et al. 2012); orphan taxa in environmental sampling databases (Nilsson et al. 2016); nomenclatural issues (Hawksworth 2011); an exhaustive checklist (Nimis et al. 2018); large scale phylogenies at family and generic levels (Miettinen et al. 2016; Plata et al. 2013); diversity of plant-associated fungi (Tibpromma et al. 2018; Yang et al. 2018); and a fungus isolated from Bison dung (Callaghan et al. 2015).

All nomenclatural changes in the journal are indexed in MycoBank. Since its launch, 1108 new species, 71 new genera and four new families have been described in MycoKeys. In addition, 248 new combinations of taxa have been proposed in the journal.

When the journal received its first Journal Impact Factor in 2015, it was at 1.846 and has subsequently increased to the current 3.3, demonstrating the quality of the peer review of submitted manuscripts, stringent quality control and management of manuscripts. The current CiteScore – a journal-level citation metric by Scopus – of MycoKeys is 5.8. Although the journal is currently in the Q2 Mycology quartile of the Web of Science, it is in the Q1 quartile in all three Scopus categories: Agricultural and Biological Sciences; Ecology, Evolution, Behavior and Systematics; and Plant Science.

MycoKeys is also active in popularizing research on social media via its own channels on X and Facebook, where updates about the most recent publications and news from the journal are currently shared to approximately 1,500 and 2,200 followers, respectively. As a result of regular press campaigns, over the years, studies published in MycoKeys have been publicized in major news media outlets, such as The Washington Post, CNN, Newsweek and Spiegel.

In its short history, MycoKeys has already played a vital role in contributing to the understanding of the evolution, diversity and taxonomy of fungi. Exciting new methods provide further insights and allow us to address questions we could not dream of a few decades ago.

## ﻿Additional information

### Author ORCIDs

H. Thorsten Lumbsch https://orcid.org/0000-0003-1512-835X

Pavel Stoev https://orcid.org/0000-0002-5702-5677

Lyubomir Penev https://orcid.org/0000-0002-2186-5033

### Data availability

All of the data that support the findings of this study are available in the main text.
